# Supplementary material for: Structural Basis of a Novel Agonistic Anti-OX40 Antibody
Source: Biomolecules. 2022 Aug 31;12(9):1209. doi: 10.3390/biom12091209 (PMC9496217; doi:10.3390/biom12091209)
Supplement: Supplementary file 1 [file biomolecules-12-01209-s001.zip › biomolecules-1877544-supplementary material and structure files/biomolecules-1877544-structure files/DF004-OX40-validation report.pdf]

Full wwPDB X-ray Structure Validation Report (i)

Aug 3, 2022 – 10:56 pm BST

PDB ID	:	8AG1
Title	:	Crystal structure of a novel OX40 antibody
Deposited on	:	2022-07-19
Resolution	:	3.30 Å(reported)

This wwPDB validation report is for manuscript review

For Manuscript Review

This is a Full wwPDB X-ray Structure Validation Report.

This report is produced by the wwPDB biocuration pipeline after annotation of the structure.

We welcome your comments at *validation@mail.wwpdb.org*
A user guide is available at
https://www.wwpdb.org/validation/2017/XrayValidationReportHelp
with specific help available everywhere you see the (i) symbol.

The types of validation reports are described at http://www.wwpdb.org/validation/2017/FAQs#types.

The following versions of software and data (see references (i)) were used in the production of this report:

MolProbity	:	4.02b-467
Mogul	:	1.8.4, CSD as541be (2020)
Xtriage (Phenix)	:	1.13
EDS	:	2.29
Percentile statistics	:	20191225.v01 (using entries in the PDB archive December 25th 2019)
Refmac	:	5.8.0267
CCP4	:	7.1.010 (Gargrove)
Ideal geometry (proteins)	:	Engh & Huber (2001)
Ideal geometry (DNA, RNA)	:	Parkinson et al. (1996)
Validation Pipeline (wwPDB-VP)	:	2.29

1 Overall quality at a glance (i)

The following experimental techniques were used to determine the structure:
X-RAY DIFFRACTION

The reported resolution of this entry is 3.30 Å.

Percentile scores (ranging between 0-100) for global validation metrics of the entry are shown in the following graphic. The table shows the number of entries on which the scores are based.

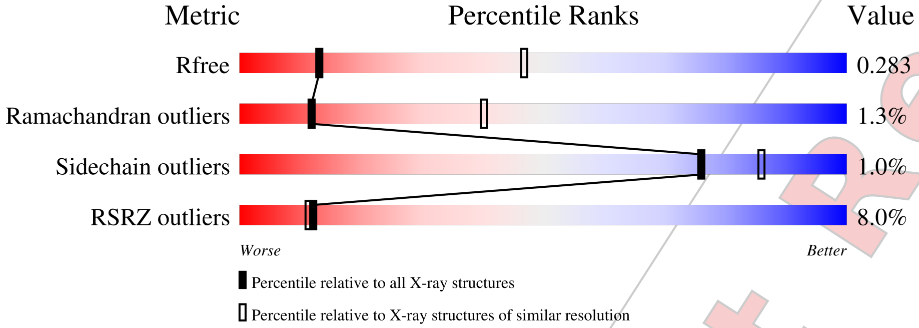

Metric	Whole archive (#Entries)	Similar resolution (#Entries, resolution range(Å))
R_{free}	130704	1149 (3.34-3.26)
Ramachandran outliers	138981	1183 (3.34-3.26)
Sidechain outliers	138945	1182 (3.34-3.26)
RSRZ outliers	127900	1115 (3.34-3.26)

The table below summarises the geometric issues observed across the polymeric chains and their fit to the electron density. The red, orange, yellow and green segments of the lower bar indicate the fraction of residues that contain outliers for >=3, 2, 1 and 0 types of geometric quality criteria respectively. A grey segment represents the fraction of residues that are not modelled. The numeric value for each fraction is indicated below the corresponding segment, with a dot representing fractions <=5% The upper red bar (where present) indicates the fraction of residues that have poor fit to the electron density. The numeric value is given above the bar.

Mol	Chain	Length	Quality of chain
1	L	216	% / 97% •
1	l	216	8% / 97% •
2	H	240	2% / 90% • 9%
2	h	240	10% / 89% • 10%
3	A	156	7% / 86% • 12%
3	a	156	17% / 45% • 53%

For Manuscript Review

2 Entry composition (i)

There are 5 unique types of molecules in this entry. The entry contains 8038 atoms, of which 0 are hydrogens and 0 are deuteriums.

In the tables below, the ZeroOcc column contains the number of atoms modelled with zero occupancy, the AltConf column contains the number of residues with at least one atom in alternate conformation and the Trace column contains the number of residues modelled with at most 2 atoms.

- Molecule 1 is a protein called Novel OX40 antibody light chain.

Mol	Chain	Residues	Atoms					ZeroOcc	AltConf	Trace
1	L	215	Total 1637	C 1024	N 277	O 332	S 4	0	1	0
1	l	216	Total 1636	C 1026	N 273	O 332	S 5	0	1	0

- Molecule 2 is a protein called Novel OX40 antibody heavy chain.

Mol	Chain	Residues	Atoms					ZeroOcc	AltConf	Trace
2	H	218	Total 1657	C 1048	N 275	O 330	S 4	0	0	0
2	h	215	Total 1631	C 1033	N 271	O 323	S 4	0	0	0

- Molecule 3 is a protein called Tumor necrosis factor receptor superfamily member 4.

Mol	Chain	Residues	Atoms					ZeroOcc	AltConf	Trace
3	A	138	Total 973	C 584	N 177	O 194	S 18	0	0	0
3	a	73	Total 488	C 295	N 84	O 97	S 12	0	0	0

There are 30 discrepancies between the modelled and reference sequences:

Chain	Residue	Modelled	Actual	Comment	Reference
A	52	LEU	MET	conflict	UNP P43489
A	171	MET	-	expression tag	UNP P43489
A	172	ASP	-	expression tag	UNP P43489
A	173	PRO	-	expression tag	UNP P43489
A	174	GLY	-	expression tag	UNP P43489
A	175	GLY	-	expression tag	UNP P43489
A	176	SER	-	expression tag	UNP P43489
A	177	HIS	-	expression tag	UNP P43489

Continued on next page...

For Manuscript Review

Continued from previous page...

Chain	Residue	Modelled	Actual	Comment	Reference
A	178	HIS	-	expression tag	UNP P43489
A	179	HIS	-	expression tag	UNP P43489
A	180	HIS	-	expression tag	UNP P43489
A	181	HIS	-	expression tag	UNP P43489
A	182	HIS	-	expression tag	UNP P43489
A	183	HIS	-	expression tag	UNP P43489
A	184	HIS	-	expression tag	UNP P43489
a	52	LEU	MET	conflict	UNP P43489
a	171	MET	-	expression tag	UNP P43489
a	172	ASP	-	expression tag	UNP P43489
a	173	PRO	-	expression tag	UNP P43489
a	174	GLY	-	expression tag	UNP P43489
a	175	GLY	-	expression tag	UNP P43489
a	176	SER	-	expression tag	UNP P43489
a	177	HIS	-	expression tag	UNP P43489
a	178	HIS	-	expression tag	UNP P43489
a	179	HIS	-	expression tag	UNP P43489
a	180	HIS	-	expression tag	UNP P43489
a	181	HIS	-	expression tag	UNP P43489
a	182	HIS	-	expression tag	UNP P43489
a	183	HIS	-	expression tag	UNP P43489
a	184	HIS	-	expression tag	UNP P43489

- Molecule 4 is 2-acetamido-2-deoxy-beta-D-glucopyranose (three-letter code: NAG) (formula: $C_8H_{15}NO_6$).

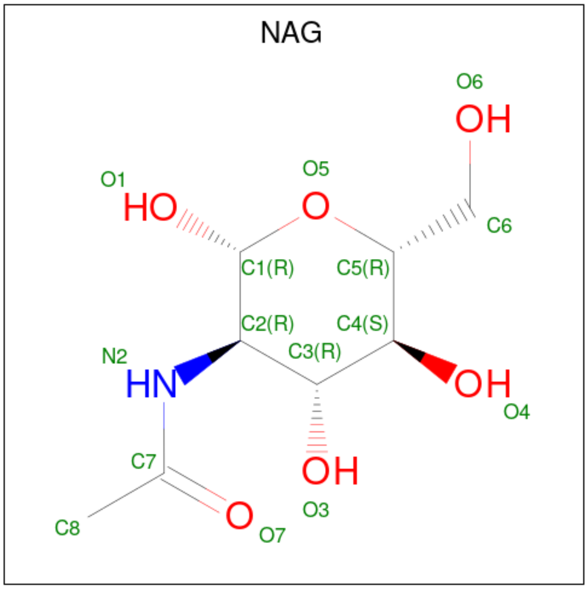

For Manuscript Review

Mol	Chain	Residues	Atoms				ZeroOcc	AltConf
			Total	C	N	O		
4	A	1	14	8	1	5	0	0

- Molecule 5 is water.

Mol	Chain	Residues	Atoms		ZeroOcc	AltConf
			Total	O		
5	L	1	1	1	0	0
5	A	1	1	1	0	0

For Manuscript Review

3 Residue-property plots (i)

These plots are drawn for all protein, RNA, DNA and oligosaccharide chains in the entry. The first graphic for a chain summarises the proportions of the various outlier classes displayed in the second graphic. The second graphic shows the sequence view annotated by issues in geometry and electron density. Residues are color-coded according to the number of geometric quality criteria for which they contain at least one outlier: green = 0, yellow = 1, orange = 2 and red = 3 or more. A red dot above a residue indicates a poor fit to the electron density (RSRZ > 2). Stretches of 2 or more consecutive residues without any outlier are shown as a green connector. Residues present in the sample, but not in the model, are shown in grey.

- Molecule 1: Novel OX40 antibody light chain

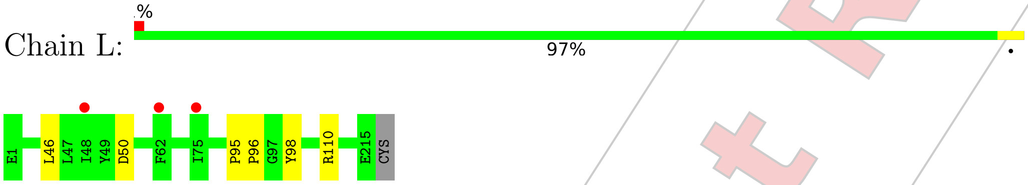

- Molecule 1: Novel OX40 antibody light chain

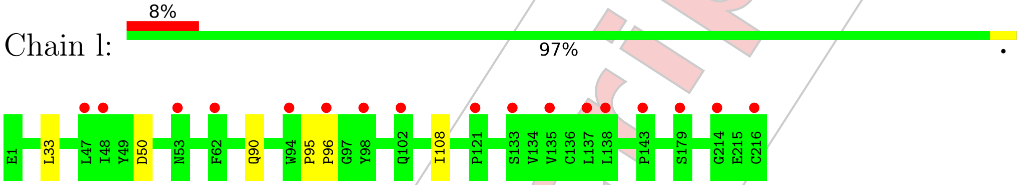

- Molecule 2: Novel OX40 antibody heavy chain

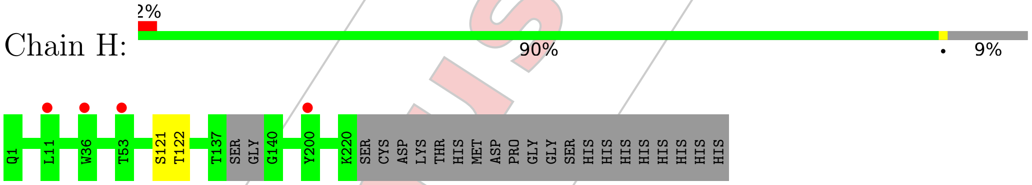

- Molecule 2: Novel OX40 antibody heavy chain

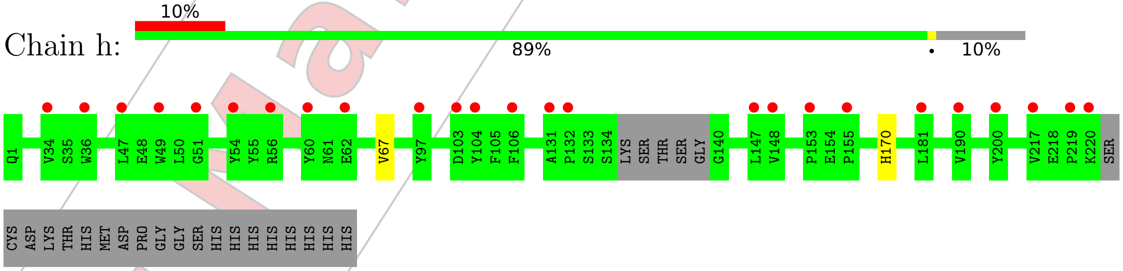

- Molecule 3: Tumor necrosis factor receptor superfamily member 4

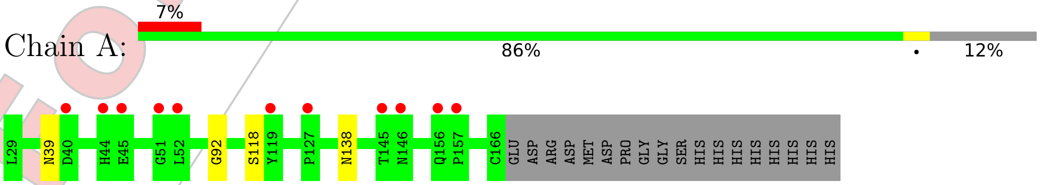

For Manuscript Review

- Molecule 3: Tumor necrosis factor receptor superfamily member 4

Chain a:

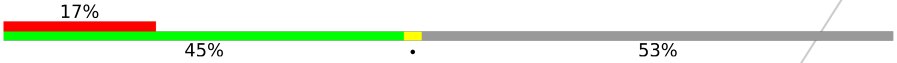

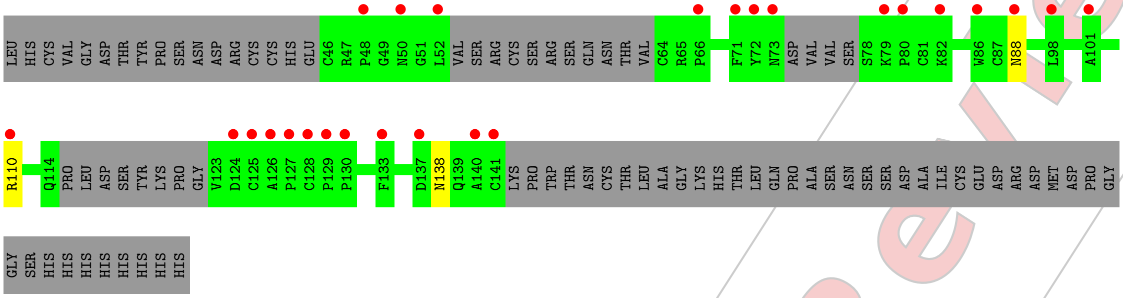

For Manuscript Review

4 Data and refinement statistics (i)

Property	Value	Source
Space group	P 62 2 2	Depositor
Cell constants<br>a, b, c, α, β, γ	162.36Å 162.36Å 232.15Å<br>90.00° 90.00° 120.00°	Depositor
Resolution (Å)	77.50 – 3.30<br>77.38 – 3.30	Depositor<br>EDS
% Data completeness<br>(in resolution range)	80.1 (77.50-3.30)<br>80.2 (77.38-3.30)	Depositor<br>EDS
R_{merge}	0.17	Depositor
R_{sym}	(Not available)	Depositor
$< I/\sigma(I) >$ [1]	2.54 (at 3.33Å)	Xtriage
Refinement program	REFMAC 8.0.002	Depositor
R, R_{free}	0.194 , 0.268<br>0.210 , 0.283	Depositor<br>DCC
R_{free} test set	1132 reflections (5.08%)	wwPDB-VP
Wilson B-factor (Å^2)	97.9	Xtriage
Anisotropy	0.006	Xtriage
Bulk solvent k_{sol}(e/Å^3), B_{sol}(Å^2)	(Not available) , (Not available)	EDS
L-test for twinning[2]	$< \|L\| > = 0.47$, $< L^2 > = 0.29$	Xtriage
Estimated twinning fraction	No twinning to report.	Xtriage
F_o,F_c correlation	0.87	EDS
Total number of atoms	8038	wwPDB-VP
Average B, all atoms (Å^2)	98.0	wwPDB-VP

Xtriage's analysis on translational NCS is as follows: *The largest off-origin peak in the Patterson function is 2.43% of the height of the origin peak. No significant pseudotranslation is detected.*

[1]Intensities estimated from amplitudes.

[2]Theoretical values of $< |L| >$, $< L^2 >$ for acentric reflections are 0.5, 0.333 respectively for untwinned datasets, and 0.375, 0.2 for perfectly twinned datasets.

For Manuscript Review

5 Model quality (i)

5.1 Standard geometry (i)

Bond lengths and bond angles in the following residue types are not validated in this section: NAG

The Z score for a bond length (or angle) is the number of standard deviations the observed value is removed from the expected value. A bond length (or angle) with $|Z| > 5$ is considered an outlier worth inspection. RMSZ is the root-mean-square of all Z scores of the bond lengths (or angles).

Mol	Chain	Bond lengths		Bond angles	
		RMSZ	#$\|Z\|$ >5	RMSZ	#$\|Z\|$ >5
1	L	0.28	0/1678	0.45	0/2287
1	l	0.28	0/1677	0.44	0/2285
2	H	0.28	0/1699	0.48	0/2325
2	h	0.28	0/1673	0.46	0/2291
3	A	0.28	0/998	0.46	0/1370
3	a	0.33	0/499	0.48	0/681
All	All	0.28	0/8224	0.46	0/11239

There are no bond length outliers.

There are no bond angle outliers.

There are no chirality outliers.

There are no planarity outliers.

5.2 Too-close contacts (i)

Due to software issues we are unable to calculate clashes - this section is therefore empty.

5.3 Torsion angles (i)

5.3.1 Protein backbone (i)

In the following table, the Percentiles column shows the percent Ramachandran outliers of the chain as a percentile score with respect to all X-ray entries followed by that with respect to entries of similar resolution.

The Analysed column shows the number of residues for which the backbone conformation was analysed, and the total number of residues.

For Manuscript Review

Mol	Chain	Analysed	Favoured	Allowed	Outliers	Percentiles	
1	L	214/216 (99%)	191 (89%)	19 (9%)	4 (2%)	8	34
1	l	215/216 (100%)	189 (88%)	23 (11%)	3 (1%)	11	38
2	H	214/240 (89%)	190 (89%)	22 (10%)	2 (1%)	17	48
2	h	211/240 (88%)	192 (91%)	19 (9%)	0	100	100
3	A	136/156 (87%)	121 (89%)	12 (9%)	3 (2%)	6	30
3	a	65/156 (42%)	55 (85%)	8 (12%)	2 (3%)	4	23
All	All	1055/1224 (86%)	938 (89%)	103 (10%)	14 (1%)	12	40

All (14) Ramachandran outliers are listed below:

Mol	Chain	Res	Type
2	H	121	SER
1	l	50	ASP
3	A	39	ASN
1	L	50	ASP
3	a	138	ASN
2	H	122	THR
3	A	118	SER
1	L	98	TYR
1	L	96	PRO
3	A	92	GLY
3	a	110	ARG
1	L	95	PRO
1	l	95	PRO
1	l	96	PRO

5.3.2 Protein sidechains (i)

In the following table, the Percentiles column shows the percent sidechain outliers of the chain as a percentile score with respect to all X-ray entries followed by that with respect to entries of similar resolution.

The Analysed column shows the number of residues for which the sidechain conformation was analysed, and the total number of residues.

Mol	Chain	Analysed	Rotameric	Outliers	Percentiles	
1	L	181/186 (97%)	179 (99%)	2 (1%)	73	85
1	l	181/186 (97%)	178 (98%)	3 (2%)	60	78
2	H	192/214 (90%)	192 (100%)	0	100	100

Continued on next page...

For Manuscript Review

Continued from previous page...

Mol	Chain	Analysed	Rotameric	Outliers	Percentiles	
2	h	188/214 (88%)	186 (99%)	2 (1%)	73	85
3	A	109/136 (80%)	108 (99%)	1 (1%)	78	87
3	a	52/136 (38%)	51 (98%)	1 (2%)	57	77
All	All	903/1072 (84%)	894 (99%)	9 (1%)	76	86

All (9) residues with a non-rotameric sidechain are listed below:

Mol	Chain	Res	Type
1	L	46	LEU
1	L	110	ARG
2	h	67	VAL
2	h	170	HIS
1	l	33	LEU
1	l	90	GLN
1	l	108	ILE
3	A	138	ASN
3	a	88	ASN

Sometimes sidechains can be flipped to improve hydrogen bonding and reduce clashes. All (13) such sidechains are listed below:

Mol	Chain	Res	Type
1	L	38	GLN
1	L	140	ASN
2	H	41	GLN
2	H	161	ASN
2	h	3	GLN
2	h	41	GLN
2	h	161	ASN
1	l	38	GLN
1	l	126	GLN
3	A	114	GLN
3	A	138	ASN
3	A	139	GLN
3	a	88	ASN

5.3.3 RNA ⓘ

There are no RNA molecules in this entry.

For Manuscript Review

5.4 Non-standard residues in protein, DNA, RNA chains (i)

There are no non-standard protein/DNA/RNA residues in this entry.

5.5 Carbohydrates (i)

There are no monosaccharides in this entry.

5.6 Ligand geometry (i)

1 ligand is modelled in this entry.

In the following table, the Counts columns list the number of bonds (or angles) for which Mogul statistics could be retrieved, the number of bonds (or angles) that are observed in the model and the number of bonds (or angles) that are defined in the Chemical Component Dictionary. The Link column lists molecule types, if any, to which the group is linked. The Z score for a bond length (or angle) is the number of standard deviations the observed value is removed from the expected value. A bond length (or angle) with $|Z| > 2$ is considered an outlier worth inspection. RMSZ is the root-mean-square of all Z scores of the bond lengths (or angles).

Mol	Type	Chain	Res	Link	Bond lengths			Bond angles		
					Counts	RMSZ	$\#\|Z\| > 2$	Counts	RMSZ	$\#\|Z\| > 2$
4	NAG	A	201	3	14,14,15	0.38	0	17,19,21	0.59	0

In the following table, the Chirals column lists the number of chiral outliers, the number of chiral centers analysed, the number of these observed in the model and the number defined in the Chemical Component Dictionary. Similar counts are reported in the Torsion and Rings columns. '-' means no outliers of that kind were identified.

Mol	Type	Chain	Res	Link	Chirals	Torsions	Rings
4	NAG	A	201	3	-	2/6/23/26	0/1/1/1

There are no bond length outliers.

There are no bond angle outliers.

There are no chirality outliers.

All (2) torsion outliers are listed below:

Mol	Chain	Res	Type	Atoms
4	A	201	NAG	C8-C7-N2-C2
4	A	201	NAG	O7-C7-N2-C2

There are no ring outliers.

For Manuscript Review

No monomer is involved in short contacts.

5.7 Other polymers (i)

There are no such residues in this entry.

5.8 Polymer linkage issues (i)

There are no chain breaks in this entry.

For Manuscript Review

6 Fit of model and data (i)

6.1 Protein, DNA and RNA chains (i)

In the following table, the column labelled ‘#RSRZ> 2’ contains the number (and percentage) of RSRZ outliers, followed by percent RSRZ outliers for the chain as percentile scores relative to all X-ray entries and entries of similar resolution. The OWAB column contains the minimum, median, 95th percentile and maximum values of the occupancy-weighted average B-factor per residue. The column labelled ‘Q< 0.9’ lists the number of (and percentage) of residues with an average occupancy less than 0.9.

Mol	Chain	Analysed	<RSRZ>	#RSRZ>2			OWAB(Å^2)	Q<0.9
1	L	215/216 (99%)	0.52	3 (1%)	75	75	53, 80, 105, 117	0
1	l	216/216 (100%)	0.51	17 (7%)	12	12	72, 120, 149, 174	0
2	H	218/240 (90%)	0.43	4 (1%)	68	67	57, 84, 119, 138	0
2	h	215/240 (89%)	0.84	25 (11%)	4	4	66, 91, 129, 159	0
3	A	138/156 (88%)	0.54	11 (7%)	12	11	66, 94, 132, 150	0
3	a	73/156 (46%)	1.61	26 (35%)	0	0	90, 130, 150, 151	2 (2%)
All	All	1075/1224 (87%)	0.64	86 (8%)	12	11	53, 95, 140, 174	2 (0%)

All (86) RSRZ outliers are listed below:

Mol	Chain	Res	Type	RSRZ
1	l	216	CYS	16.3
3	a	141	CYS	6.0
3	a	129	PRO	5.2
2	h	131	ALA	5.1
3	a	140	ALA	5.1
3	a	110	ARG	5.0
2	h	60	TYR	4.3
3	a	128	CYS	4.1
3	a	124	ASP	4.0
3	a	137	ASP	4.0
3	a	73	ASN	3.9
3	A	146	ASN	3.8
1	l	98	TYR	3.6
3	a	80	PRO	3.6
2	h	106	PHE	3.6
1	l	47	LEU	3.5
3	A	51	GLY	3.5
3	a	133	PHE	3.4
1	L	62	PHE	3.3

Continued on next page...

For Manuscript Review

Continued from previous page...

Mol	Chain	Res	Type	RSRZ
1	l	62	PHE	3.3
3	a	127	PRO	3.2
3	a	126	ALA	3.2
3	a	48	PRO	3.1
2	h	220	LYS	3.1
2	h	34	VAL	3.1
2	h	51	GLY	3.1
3	a	79	LYS	3.1
3	A	40	ASP	3.0
2	h	190	VAL	3.0
2	h	49	TRP	3.0
3	A	156	GLN	3.0
3	A	119	TYR	2.9
3	a	86	TRP	2.9
1	l	121	PRO	2.9
2	h	104	TYR	2.9
3	a	50	ASN	2.9
1	l	102	GLN	2.8
1	l	96	PRO	2.8
1	L	75	ILE	2.8
3	a	71	PHE	2.8
2	h	217	VAL	2.7
3	A	44	HIS	2.7
3	a	72	TYR	2.7
1	l	94	TRP	2.7
2	h	103	ASP	2.7
2	h	36	TRP	2.7
1	l	137	LEU	2.7
2	h	219	PRO	2.6
1	l	135	VAL	2.6
2	h	97	TYR	2.6
3	a	130	PRO	2.5
3	A	145	THR	2.5
2	h	155	PRO	2.5
1	l	53	ASN	2.5
3	a	82	LYS	2.4
2	h	153	PRO	2.4
2	H	36	TRP	2.4
2	h	147	LEU	2.4
3	A	52	LEU	2.4
2	h	54	TYR	2.3
2	h	181	LEU	2.3

Continued on next page...

For Manuscript Review

Continued from previous page...

Mol	Chain	Res	Type	RSRZ
3	a	101	ALA	2.3
3	a	52	LEU	2.3
1	l	133	SER	2.3
1	l	48	ILE	2.3
1	l	214	GLY	2.3
3	A	45	GLU	2.3
1	l	138	LEU	2.2
1	l	179	SER	2.2
2	h	200	TYR	2.2
3	a	66	PRO	2.2
3	a	88	ASN	2.2
2	h	132	PRO	2.2
3	A	157	PRO	2.1
1	L	48	ILE	2.1
2	h	62	GLU	2.1
2	h	148	VAL	2.1
2	h	56	ARG	2.1
1	l	143	PRO	2.1
2	H	200	TYR	2.0
3	A	127	PRO	2.0
3	a	98	LEU	2.0
3	a	125	CYS	2.0
2	h	47	LEU	2.0
2	H	53	THR	2.0
2	H	11	LEU	2.0

6.2 Non-standard residues in protein, DNA, RNA chains (i)

There are no non-standard protein/DNA/RNA residues in this entry.

6.3 Carbohydrates (i)

There are no monosaccharides in this entry.

6.4 Ligands (i)

In the following table, the Atoms column lists the number of modelled atoms in the group and the number defined in the chemical component dictionary. The B-factors column lists the minimum, median, 95th percentile and maximum values of B factors of atoms in the group. The column labelled 'Q< 0.9' lists the number of atoms with occupancy less than 0.9.

For Manuscript Review

Mol	Type	Chain	Res	Atoms	RSCC	RSR	B-factors($Å^2$)	Q<0.9
4	NAG	A	201	14/15	0.85	0.17	88,107,115,116	0

6.5 Other polymers ⓘ

There are no such residues in this entry.

For Manuscript Review
