# Supplementary material for: Structural Basis of a Novel Agonistic Anti-OX40 Antibody
Source: Biomolecules. 2022 Aug 31;12(9):1209. doi: 10.3390/biom12091209 (PMC9496217; doi:10.3390/biom12091209)
Supplement: Supplementary file 1 [file biomolecules-12-01209-s001.zip › biomolecules-1877544-supplementary material and structure files/biomolecules-1877544-structure files/OX40-7888_val-report-full_P1-3.pdf]

Full wwPDB X-ray Structure Validation Report (i)

Jul 22, 2022 – 11:58 AM JST

PDB ID	:	7YK4
Title	:	ox40-antibody
Deposited on	:	2022-07-21
Resolution	:	2.70 Å(reported)

This wwPDB validation report is for manuscript review

This is a Full wwPDB X-ray Structure Validation Report.

This report is produced by the wwPDB biocuration pipeline after annotation of the structure.

We welcome your comments at *validation@mail.wwpdb.org*
A user guide is available at
https://www.wwpdb.org/validation/2017/XrayValidationReportHelp
with specific help available everywhere you see the (i) symbol.

The types of validation reports are described at http://www.wwpdb.org/validation/2017/FAQs#types.

The following versions of software and data (see references (i)) were used in the production of this report:

MolProbity	:	4.02b-467
Mogul	:	?.? (???), CSD ??CSD?? (????)
Xtriage (Phenix)	:	1.13
EDS	:	2.29
buster-report	:	1.1.7 (2018)
Percentile statistics	:	20191225.v01 (using entries in the PDB archive December 25th 2019)
Refmac	:	5.8.0158
CCP4	:	7.0.044 (Gargrove)
Ideal geometry (proteins)	:	Engh & Huber (2001)
Ideal geometry (DNA, RNA)	:	Parkinson et al. (1996)
Validation Pipeline (wwPDB-VP)	:	2.29

For Manuscript Review

1 Overall quality at a glance (i)

The following experimental techniques were used to determine the structure:
X-RAY DIFFRACTION

The reported resolution of this entry is 2.70 Å.

Percentile scores (ranging between 0-100) for global validation metrics of the entry are shown in the following graphic. The table shows the number of entries on which the scores are based.

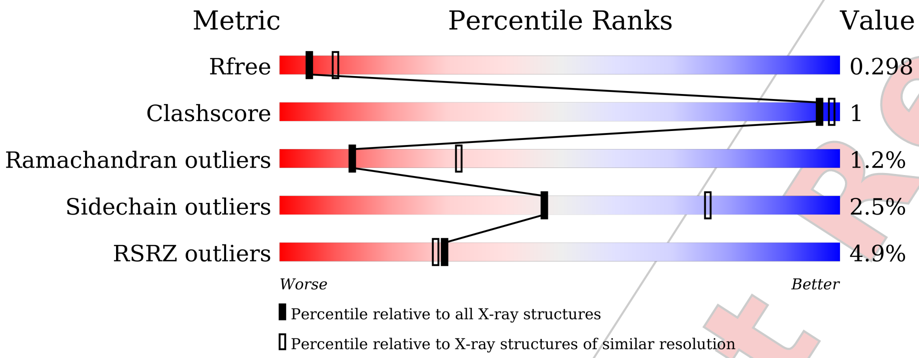

Metric	Whole archive (#Entries)	Similar resolution (#Entries, resolution range(Å))
R_{free}	130704	2808 (2.70-2.70)
Clashscore	141614	3122 (2.70-2.70)
Ramachandran outliers	138981	3069 (2.70-2.70)
Sidechain outliers	138945	3069 (2.70-2.70)
RSRZ outliers	127900	2737 (2.70-2.70)

The table below summarises the geometric issues observed across the polymeric chains and their fit to the electron density. The red, orange, yellow and green segments of the lower bar indicate the fraction of residues that contain outliers for >=3, 2, 1 and 0 types of geometric quality criteria respectively. A grey segment represents the fraction of residues that are not modelled. The numeric value for each fraction is indicated below the corresponding segment, with a dot representing fractions <=5% The upper red bar (where present) indicates the fraction of residues that have poor fit to the electron density. The numeric value is given above the bar.

Mol	Chain	Length	Quality of chain
1	H	238	% / 87% • 9%
1	I	238	3% / 84% 7% 9%
2	L	214	% / 92% 7% •
2	M	214	3% / 96% • •
3	A	156	13% / 47% • 51%

Continued on next page...

For Manuscript Review

Continued from previous page...

Mol	Chain	Length	Quality of chain
3	B	156	5% 31% • 65%

For Manuscript Review

2 Entry composition (i)

There are 5 unique types of molecules in this entry. The entry contains 7418 atoms, of which 0 are hydrogens and 0 are deuteriums.

In the tables below, the ZeroOcc column contains the number of atoms modelled with zero occupancy, the AltConf column contains the number of residues with at least one atom in alternate conformation and the Trace column contains the number of residues modelled with at most 2 atoms.

- Molecule 1 is a protein called antibody-H.

Mol	Chain	Residues	Atoms	ZeroOcc	AltConf	Trace
1	H	216	Total C N O S<br>1625 1026 268 325 6	0	0	0
1	I	216	Total C N O S<br>1609 1018 261 324 6	0	0	0

- Molecule 2 is a protein called antibody-L.

Mol	Chain	Residues	Atoms	ZeroOcc	AltConf	Trace
2	L	212	Total C N O S<br>1638 1024 279 330 5	0	0	0
2	M	211	Total C N O S<br>1616 1010 272 329 5	0	0	0

- Molecule 3 is a protein called Tumor necrosis factor receptor superfamily member 4.

Mol	Chain	Residues	Atoms	ZeroOcc	AltConf	Trace
3	A	77	Total C N O S<br>535 319 96 110 10	0	0	0
3	B	54	Total C N O S<br>380 229 69 74 8	0	0	0

There are 30 discrepancies between the modelled and reference sequences:

Chain	Residue	Modelled	Actual	Comment	Reference
A	52	LEU	MET	conflict	UNP P43489
A	171	MET	-	expression tag	UNP P43489
A	172	ASP	-	expression tag	UNP P43489
A	173	PRO	-	expression tag	UNP P43489
A	174	GLY	-	expression tag	UNP P43489
A	175	GLY	-	expression tag	UNP P43489
A	176	SER	-	expression tag	UNP P43489
A	177	HIS	-	expression tag	UNP P43489

Continued on next page...

For Manuscript Review

Continued from previous page...

Chain	Residue	Modelled	Actual	Comment	Reference
A	178	HIS	-	expression tag	UNP P43489
A	179	HIS	-	expression tag	UNP P43489
A	180	HIS	-	expression tag	UNP P43489
A	181	HIS	-	expression tag	UNP P43489
A	182	HIS	-	expression tag	UNP P43489
A	183	HIS	-	expression tag	UNP P43489
A	184	HIS	-	expression tag	UNP P43489
B	52	LEU	MET	conflict	UNP P43489
B	171	MET	-	expression tag	UNP P43489
B	172	ASP	-	expression tag	UNP P43489
B	173	PRO	-	expression tag	UNP P43489
B	174	GLY	-	expression tag	UNP P43489
B	175	GLY	-	expression tag	UNP P43489
B	176	SER	-	expression tag	UNP P43489
B	177	HIS	-	expression tag	UNP P43489
B	178	HIS	-	expression tag	UNP P43489
B	179	HIS	-	expression tag	UNP P43489
B	180	HIS	-	expression tag	UNP P43489
B	181	HIS	-	expression tag	UNP P43489
B	182	HIS	-	expression tag	UNP P43489
B	183	HIS	-	expression tag	UNP P43489
B	184	HIS	-	expression tag	UNP P43489

- Molecule 4 is 2-acetamido-2-deoxy-beta-D-glucopyranose (three-letter code: NAG) (formula: $C_8H_{15}NO_6$) (labeled as "Ligand of Interest" by depositor).

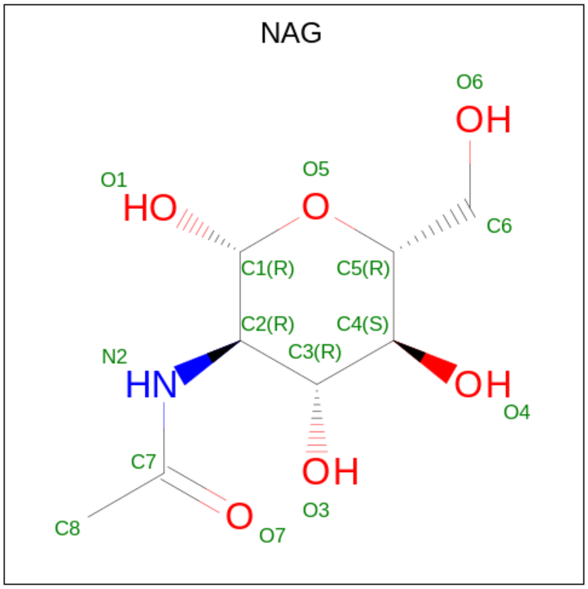

For Manuscript Review

Mol	Chain	Residues	Atoms				ZeroOcc	AltConf
			Total	C	N	O		
4	A	1	14	8	1	5	0	0

- Molecule 5 is water.

Mol	Chain	Residues	Atoms		ZeroOcc	AltConf
			Total	O		
5	L	1	1	1	0	0

For Manuscript Review

3 Residue-property plots (i)

These plots are drawn for all protein, RNA, DNA and oligosaccharide chains in the entry. The first graphic for a chain summarises the proportions of the various outlier classes displayed in the second graphic. The second graphic shows the sequence view annotated by issues in geometry and electron density. Residues are color-coded according to the number of geometric quality criteria for which they contain at least one outlier: green = 0, yellow = 1, orange = 2 and red = 3 or more. A red dot above a residue indicates a poor fit to the electron density (RSRZ > 2). Stretches of 2 or more consecutive residues without any outlier are shown as a green connector. Residues present in the sample, but not in the model, are shown in grey.

- Molecule 1: antibody-H

Chain H:

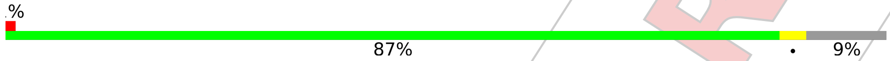

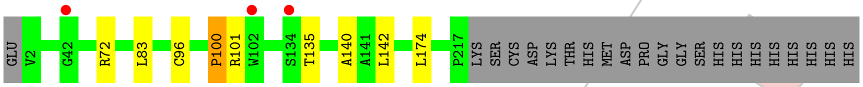

- Molecule 1: antibody-H

Chain I:

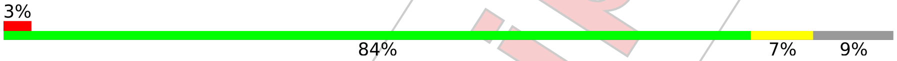

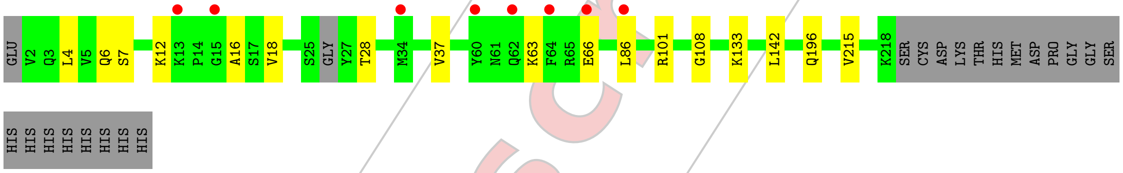

- Molecule 2: antibody-L

Chain L:

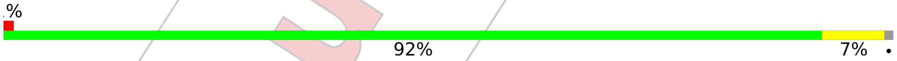

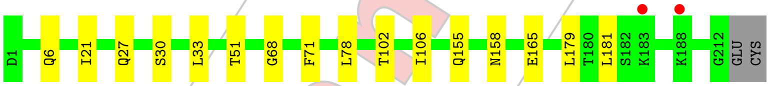

- Molecule 2: antibody-L

Chain M:

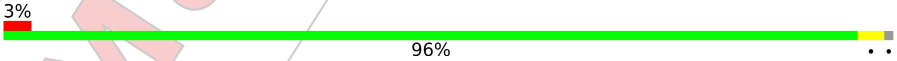

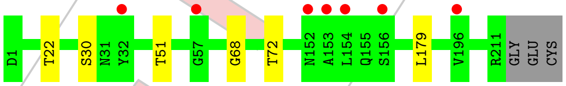

- Molecule 3: Tumor necrosis factor receptor superfamily member 4

Chain A:

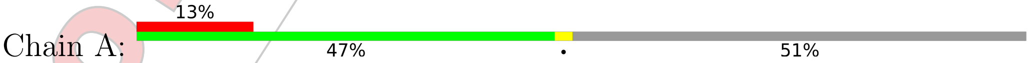

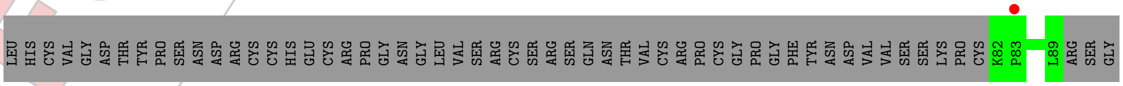

For Manuscript Review

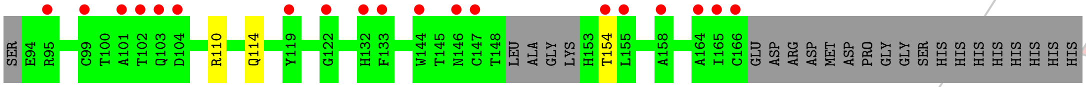

• Molecule 3: Tumor necrosis factor receptor superfamily member 4

Chain B:
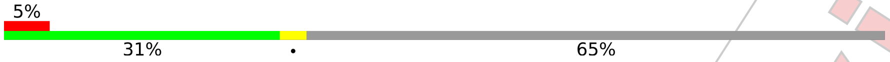

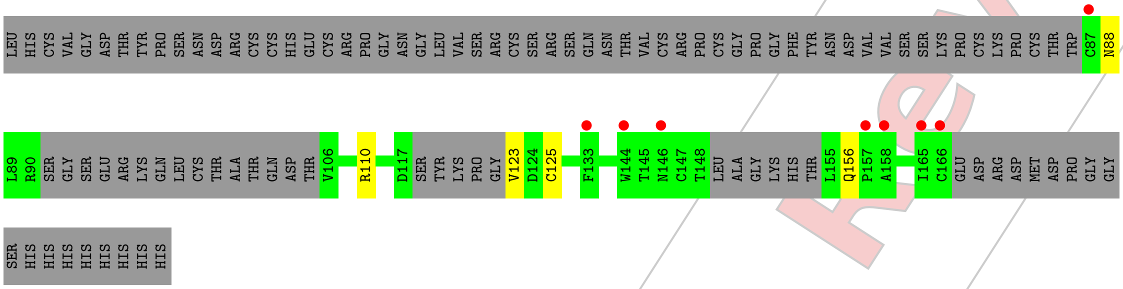

For Manuscript Review

4 Data and refinement statistics (i)

Property	Value	Source
Space group	P 43 21 2	Depositor
Cell constants<br>a, b, c, α, β, γ	124.77Å 124.77Å 194.90Å<br>90.00° 90.00° 90.00°	Depositor
Resolution (Å)	48.73 – 2.70<br>48.73 – 2.70	Depositor<br>EDS
% Data completeness<br>(in resolution range)	94.0 (48.73-2.70)<br>94.0 (48.73-2.70)	Depositor<br>EDS
R_{merge}	0.15	Depositor
R_{sym}	(Not available)	Depositor
$< I/\sigma(I) >$ [1]	1.08 (at 2.69Å)	Xtriage
Refinement program	PHENIX 1.16_3549, REFMAC 1.16_3549	Depositor
R, R_{free}	0.233 , 0.298<br>0.233 , 0.298	Depositor<br>DCC
R_{free} test set	2056 reflections (5.09%)	wwPDB-VP
Wilson B-factor (Å^2)	87.8	Xtriage
Anisotropy	0.420	Xtriage
Bulk solvent k_{sol}(e/Å^3), B_{sol}(Å^2)	0.29 , 74.1	EDS
L-test for twinning[2]	$< \|L\| > = 0.47$, $< L^2 > = 0.30$	Xtriage
Estimated twinning fraction	No twinning to report.	Xtriage
F_o,F_c correlation	0.94	EDS
Total number of atoms	7418	wwPDB-VP
Average B, all atoms (Å^2)	114.0	wwPDB-VP

Xtriage's analysis on translational NCS is as follows: *The largest off-origin peak in the Patterson function is 11.47% of the height of the origin peak. No significant pseudotranslation is detected.*

[1] Intensities estimated from amplitudes.
[2] Theoretical values of $< |L| >$, $< L^2 >$ for acentric reflections are 0.5, 0.333 respectively for untwinned datasets, and 0.375, 0.2 for perfectly twinned datasets.

For Manuscript Review

5 Model quality (i)

5.1 Standard geometry (i)

Bond lengths and bond angles in the following residue types are not validated in this section: NAG

The Z score for a bond length (or angle) is the number of standard deviations the observed value is removed from the expected value. A bond length (or angle) with $|Z| > 5$ is considered an outlier worth inspection. RMSZ is the root-mean-square of all Z scores of the bond lengths (or angles).

Mol	Chain	Bond lengths		Bond angles	
		RMSZ	#$\|Z\|$ >5	RMSZ	#$\|Z\|$ >5
1	H	0.35	0/1666	0.56	0/2276
1	I	0.35	0/1648	0.55	0/2251
2	L	0.33	0/1674	0.56	0/2273
2	M	0.35	0/1652	0.55	0/2249
3	A	0.36	0/546	0.56	0/749
3	B	0.39	0/387	0.54	0/527
All	All	0.35	0/7573	0.56	0/10325

There are no bond length outliers.

There are no bond angle outliers.

There are no chirality outliers.

There are no planarity outliers.

5.2 Too-close contacts (i)

In the following table, the Non-H and H(model) columns list the number of non-hydrogen atoms and hydrogen atoms in the chain respectively. The H(added) column lists the number of hydrogen atoms added and optimized by MolProbity. The Clashes column lists the number of clashes within the asymmetric unit, whereas Symm-Clashes lists symmetry-related clashes.

Mol	Chain	Non-H	H(model)	H(added)	Clashes	Symm-Clashes
1	H	1625	0	1578	2	0
1	I	1609	0	1558	5	0
2	L	1638	0	1598	5	0
2	M	1616	0	1551	1	0
3	A	535	0	458	2	0
3	B	380	0	335	0	0
4	A	14	0	13	0	0

Continued on next page...

For Manuscript Review

Continued from previous page...

Mol	Chain	Non-H	H(model)	H(added)	Clashes	Symm-Clashes
5	L	1	0	0	0	0
All	All	7418	0	7091	14	0

The all-atom clashscore is defined as the number of clashes found per 1000 atoms (including hydrogen atoms). The all-atom clashscore for this structure is 1.

All (14) close contacts within the same asymmetric unit are listed below, sorted by their clash magnitude.

Atom-1	Atom-2	Interatomic distance (Å)	Clash overlap (Å)
2:L:21:ILE:HG23	2:L:102:THR:HG21	1.77	0.65
2:L:78:LEU:HD21	2:L:106:ILE:HD12	1.80	0.63
2:L:155:GLN:HE21	2:L:158:ASN:HD21	1.51	0.59
2:M:22:THR:HG22	2:M:72:THR:HG22	1.86	0.56
1:I:12:LYS:HG3	1:I:18:VAL:HG22	1.90	0.53
1:I:16:ALA:O	1:I:86:LEU:HD23	2.10	0.51
1:I:142:LEU:HD12	1:I:215:VAL:CG2	2.47	0.45
1:H:100:PRO:C	3:A:114:GLN:HE22	2.22	0.43
2:L:33:LEU:HD22	2:L:71:PHE:CG	2.53	0.43
3:A:154:THR:O	3:A:154:THR:HG23	2.19	0.42
2:L:6:GLN:NE2	2:L:102:THR:HG23	2.34	0.42
1:H:135:THR:HG22	1:H:140:ALA:HB2	2.01	0.41
1:I:142:LEU:HD12	1:I:215:VAL:HG23	2.01	0.41
1:I:6:GLN:HE21	1:I:108:GLY:HA3	1.85	0.41

There are no symmetry-related clashes.

5.3 Torsion angles (i)

5.3.1 Protein backbone (i)

In the following table, the Percentiles column shows the percent Ramachandran outliers of the chain as a percentile score with respect to all X-ray entries followed by that with respect to entries of similar resolution.

The Analysed column shows the number of residues for which the backbone conformation was analysed, and the total number of residues.

Mol	Chain	Analysed	Favoured	Allowed	Outliers	Percentiles	
1	H	214/238 (90%)	194 (91%)	18 (8%)	2 (1%)	17	40
1	I	212/238 (89%)	194 (92%)	16 (8%)	2 (1%)	17	40

Continued on next page...

For Manuscript Review

Continued from previous page...

Mol	Chain	Analysed	Favoured	Allowed	Outliers	Percentiles	
2	L	210/214 (98%)	195 (93%)	12 (6%)	3 (1%)	11	28
2	M	209/214 (98%)	195 (93%)	11 (5%)	3 (1%)	11	28
3	A	71/156 (46%)	64 (90%)	7 (10%)	0	100	100
3	B	46/156 (30%)	43 (94%)	1 (2%)	2 (4%)	2	5
All	All	962/1216 (79%)	885 (92%)	65 (7%)	12 (1%)	13	32

All (12) Ramachandran outliers are listed below:

Mol	Chain	Res	Type
2	L	68	GLY
1	I	101	ARG
2	M	51	THR
3	B	88	ASN
2	L	51	THR
1	I	7	SER
3	B	156	GLN
1	H	101	ARG
2	L	30	SER
2	M	30	SER
2	M	68	GLY
1	H	100	PRO

5.3.2 Protein sidechains ⓘ

In the following table, the Percentiles column shows the percent sidechain outliers of the chain as a percentile score with respect to all X-ray entries followed by that with respect to entries of similar resolution.

The Analysed column shows the number of residues for which the sidechain conformation was analysed, and the total number of residues.

Mol	Chain	Analysed	Rotameric	Outliers	Percentiles	
1	H	185/206 (90%)	180 (97%)	5 (3%)	44	74
1	I	183/206 (89%)	176 (96%)	7 (4%)	33	62
2	L	188/190 (99%)	184 (98%)	4 (2%)	53	80
2	M	184/190 (97%)	183 (100%)	1 (0%)	88	96
3	A	59/136 (43%)	58 (98%)	1 (2%)	60	84
3	B	43/136 (32%)	40 (93%)	3 (7%)	15	35

Continued on next page...

For Manuscript Review

Continued from previous page...

Mol	Chain	Analysed	Rotameric	Outliers	Percentiles	
All	All	842/1064 (79%)	821 (98%)	21 (2%)	47	76

All (21) residues with a non-rotameric sidechain are listed below:

Mol	Chain	Res	Type
1	H	72	ARG
1	H	83	LEU
1	H	96	CYS
1	H	142	LEU
1	H	174	LEU
2	L	27	GLN
2	L	165	GLU
2	L	179	LEU
2	L	181	LEU
3	A	110	ARG
1	I	4	LEU
1	I	28	THR
1	I	37	VAL
1	I	63	LYS
1	I	66	GLU
1	I	133	LYS
1	I	196	GLN
2	M	179	LEU
3	B	110	ARG
3	B	123	VAL
3	B	125	CYS

Sometimes sidechains can be flipped to improve hydrogen bonding and reduce clashes. All (6) such sidechains are listed below:

Mol	Chain	Res	Type
2	L	27	GLN
2	L	31	ASN
2	L	155	GLN
3	A	114	GLN
1	I	6	GLN
2	M	155	GLN

5.3.3 RNA ⓘ

There are no RNA molecules in this entry.

For Manuscript Review

5.4 Non-standard residues in protein, DNA, RNA chains (i)

There are no non-standard protein/DNA/RNA residues in this entry.

5.5 Carbohydrates (i)

There are no monosaccharides in this entry.

5.6 Ligand geometry (i)

1 ligand is modelled in this entry.

There are no bond length outliers.

There are no bond angle outliers.

There are no chirality outliers.

There are no torsion outliers.

There are no ring outliers.

No monomer is involved in short contacts.

The following is a two-dimensional graphical depiction of Mogul quality analysis of bond lengths, bond angles, torsion angles, and ring geometry for all instances of the Ligand of Interest. In addition, ligands with molecular weight > 250 and outliers as shown on the validation Tables will also be included. For torsion angles, if less then 5% of the Mogul distribution of torsion angles is within 10 degrees of the torsion angle in question, then that torsion angle is considered an outlier. Any bond that is central to one or more torsion angles identified as an outlier by Mogul will be highlighted in the graph. For rings, the root-mean-square deviation (RMSD) between the ring in question and similar rings identified by Mogul is calculated over all ring torsion angles. If the average RMSD is greater than 60 degrees and the minimal RMSD between the ring in question and any Mogul-identified rings is also greater than 60 degrees, then that ring is considered an outlier. The outliers are highlighted in purple. The color gray indicates Mogul did not find sufficient equivalents in the CSD to analyse the geometry.

For Manuscript Review

Ligand NAG A 201

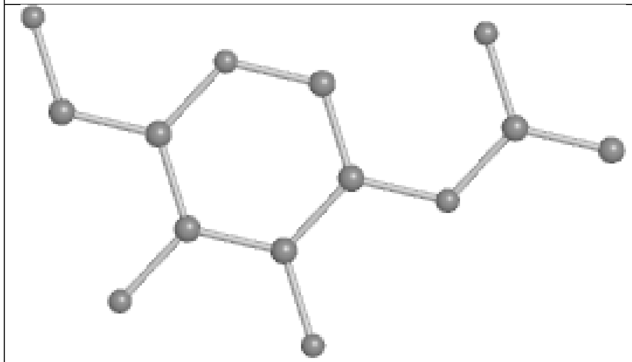

Bond lengths

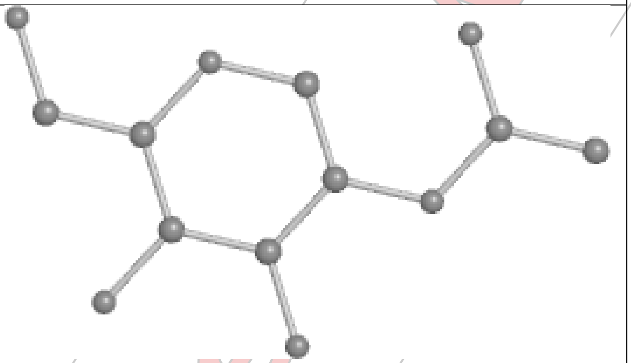

Bond angles

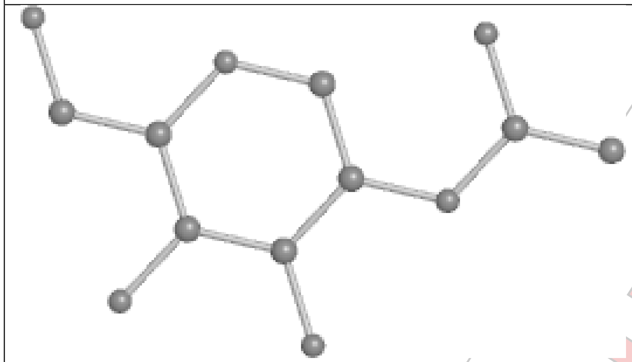

Torsions

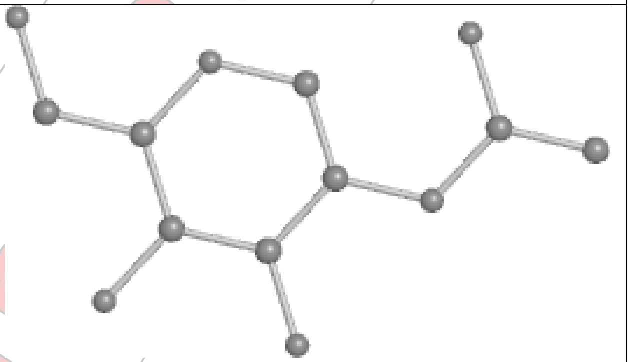

Rings

5.7 Other polymers (i)

There are no such residues in this entry.

5.8 Polymer linkage issues (i)

There are no chain breaks in this entry.

For Manuscript Review

6 Fit of model and data (i)

6.1 Protein, DNA and RNA chains (i)

In the following table, the column labelled '#RSRZ> 2' contains the number (and percentage) of RSRZ outliers, followed by percent RSRZ outliers for the chain as percentile scores relative to all X-ray entries and entries of similar resolution. The OWAB column contains the minimum, median, 95th percentile and maximum values of the occupancy-weighted average B-factor per residue. The column labelled 'Q< 0.9' lists the number of (and percentage) of residues with an average occupancy less than 0.9.

Mol	Chain	Analysed	<RSRZ>	#RSRZ>2			OWAB(Å^2)	Q<0.9
1	H	216/238 (90%)	0.14	3 (1%)	75	77	73, 98, 143, 157	0
1	I	216/238 (90%)	0.24	8 (3%)	41	41	70, 108, 161, 184	0
2	L	212/214 (99%)	0.23	2 (0%)	84	85	76, 103, 128, 152	0
2	M	211/214 (98%)	0.23	7 (3%)	46	46	74, 105, 136, 147	0
3	A	77/156 (49%)	1.08	20 (25%)	0	0	124, 163, 228, 256	0
3	B	54/156 (34%)	0.70	8 (14%)	2	1	134, 160, 223, 259	0
All	All	986/1216 (81%)	0.30	48 (4%)	29	28	70, 108, 172, 259	0

All (48) RSRZ outliers are listed below:

Mol	Chain	Res	Type	RSRZ
3	A	154	THR	4.5
3	A	95	ARG	4.2
3	A	155	LEU	4.0
3	A	122	GLY	4.0
3	A	133	PHE	3.9
2	M	196	VAL	3.6
3	B	166	CYS	3.5
1	I	62	GLN	3.3
3	B	144	TRP	3.3
3	B	157	PRO	3.2
2	M	154	LEU	3.1
1	H	42	GLY	3.1
1	I	13	LYS	3.1
3	B	87	CYS	3.0
3	B	158	ALA	3.0
3	A	83	PRO	3.0
1	H	102	TRP	2.9
1	I	86	LEU	2.8
3	A	165	ILE	2.8

Continued on next page...

For Manuscript Review

Continued from previous page...

Mol	Chain	Res	Type	RSRZ
1	I	60	TYR	2.7
2	M	152	ASN	2.7
1	I	64	PHE	2.7
3	A	102	THR	2.7
3	A	158	ALA	2.5
3	A	166	CYS	2.5
2	M	32	TYR	2.5
3	A	119	TYR	2.5
2	M	153	ALA	2.4
3	B	133	PHE	2.4
1	I	34	MET	2.3
3	A	103	GLN	2.3
3	A	104	ASP	2.3
3	A	132	HIS	2.3
2	L	188	LYS	2.3
1	H	134	SER	2.2
3	A	146	ASN	2.2
3	B	146	ASN	2.2
2	M	156	SER	2.2
3	A	101	ALA	2.2
3	A	164	ALA	2.2
3	A	144	TRP	2.2
1	I	15	GLY	2.2
2	L	183	LYS	2.2
3	B	165	ILE	2.1
1	I	66	GLU	2.1
3	A	147	CYS	2.1
3	A	99	CYS	2.0
2	M	57	GLY	2.0

6.2 Non-standard residues in protein, DNA, RNA chains ⓘ

There are no non-standard protein/DNA/RNA residues in this entry.

6.3 Carbohydrates ⓘ

There are no monosaccharides in this entry.

For Manuscript Review

6.4 Ligands ⓘ

In the following table, the Atoms column lists the number of modelled atoms in the group and the number defined in the chemical component dictionary. The B-factors column lists the minimum, median, 95th percentile and maximum values of B factors of atoms in the group. The column labelled 'Q< 0.9' lists the number of atoms with occupancy less than 0.9.

Mol	Type	Chain	Res	Atoms	RSCC	RSR	B-factors($Å^2$)	Q<0.9
4	NAG	A	201	14/15	0.86	0.13	139,152,172,177	0

The following is a graphical depiction of the model fit to experimental electron density of all instances of the Ligand of Interest. In addition, ligands with molecular weight > 250 and outliers as shown on the geometry validation Tables will also be included. Each fit is shown from different orientation to approximate a three-dimensional view.

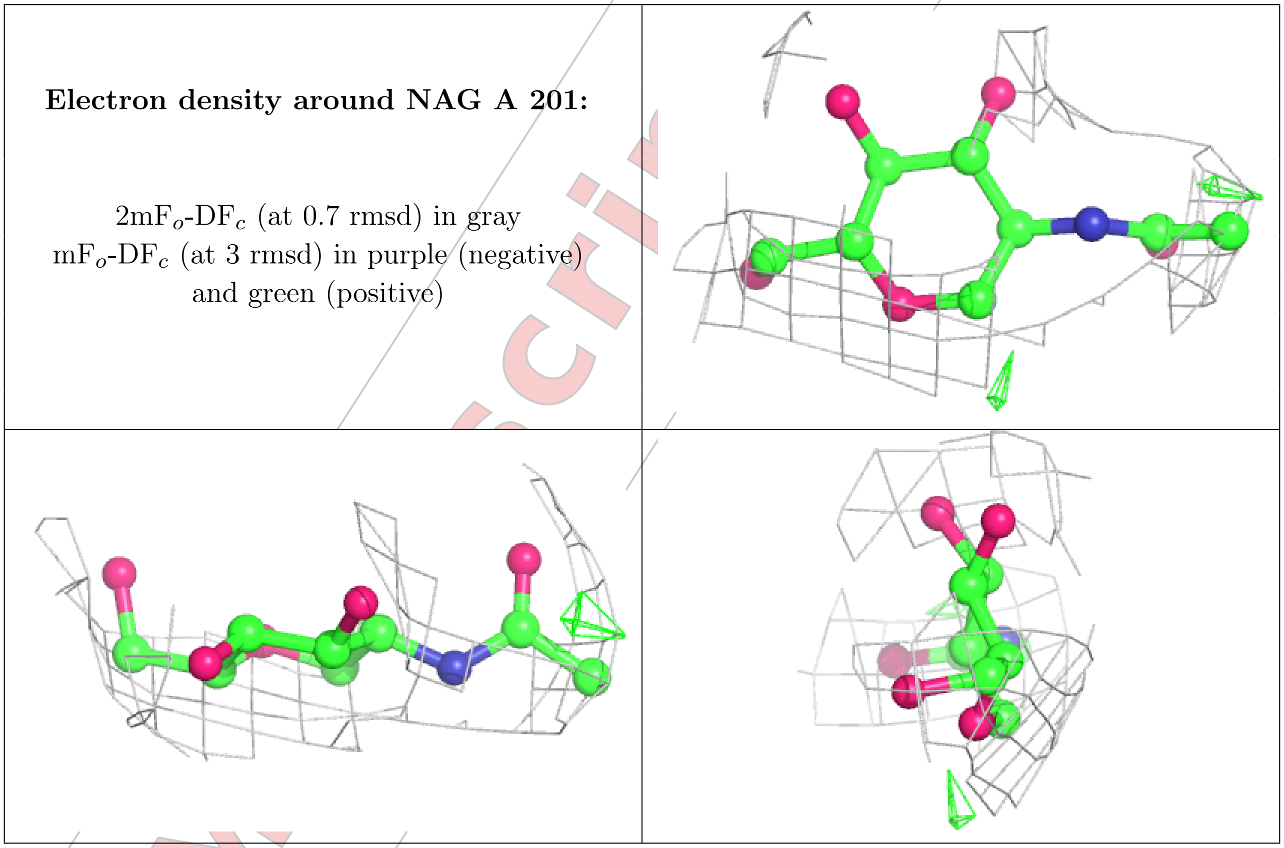

6.5 Other polymers ⓘ

There are no such residues in this entry.

For Manuscript Review
