# Supplementary material for: Structural Basis of a Novel Agonistic Anti-OX40 Antibody
Source: Biomolecules. 2022 Aug 31;12(9):1209. doi: 10.3390/biom12091209 (PMC9496217; doi:10.3390/biom12091209)
Supplement: Supplementary file 1 [file biomolecules-12-01209-s001.zip › biomolecules-1877544-supplementary material and structure files/biomolecules-1877544-supplementary-revise 1.pdf]

Supplementary Materials

Tables S1–S4

Figure S1

Supplementary Tables

Table S1. Data collection and refinement statistics.

	OX40/DF004-Fab	OX40/RG7888-Fab
Data collection		
Space group	P 6_2 2 2	P43 21 2
a, b, c (Å)	162.34, 162.34, 231.722	124.77 124.77 194.90
α, β, γ (°)	90, 90, 120	90, 90, 90
Wavelength (Å)	0.9785	0.9979
Resolution (Å)	77.38−3.30 (3.50−3.30)	48.73−2.7 (2.77−2.70)
Total NO. of observation	296081 (45757)	384382(28581)
Total NO. unique	27927 (4370)	40452(3211)
Rmerge (%)	0.168(1.945)	0.154 (0.992)
I/σI	9.1 (1.5)	9.8/1.8
Completeness (%)	99.7 (98.6)	94.14 (78.99)
Multiplicity	10.6(10.5)	9.5 (8.9)
Refinement		
Resolution (Å)	40.59−3.30 (3.50−3.30)	48.73−2.70 (2.797−2.7)
No. of reflections	27927 (4370)	40447 (3331)
No. of atoms	8055	12921
R_{work}/ R_{free}	0.1952/0.2696	0.2449/0.2971
Average B-factor	88.94	82.82
Bond length RMSD (Å)	0.017	0.006
Bond angle RMSD (°)	0.59	1.09
Ramachandran plot		
In preferred region (%)	88.89	92.00
In allowed region (%)	9.78	6.76
Outliers (%)	1.33	1.25
PDB ID	8AG1	7YK4

Table S2. Hydrogen bonds between DF004 and OX40 (distance ≤ 3.7 Å))

DF004 contact residue	DF004 Residue location	OX40 contact residue	OX40 residue location
R52	C′ strand	P69	CRD2
N102	HCDR3	P69	CRD2
Y98	LCDR3	P69	CRD2
R91	LCDR3	G70	CRD2

Table S3. Polar interactions between OX40 and RG7888-Fab (distance ≤ 3.7Å)

RG7888 contact residue	RG7888 residue location	OX40 contact residue	OX40 residue location
Y103	HCDR3	P127	CRD3
R53	C″ strand	S91	CRD2
Y49	C′ strand	D124	CRD3
Y49	C′ strand	C125	CRD3
Y32	LCDR1	C128	CRD3

Table S4. Binding surface area on OX40 contributed by DF004, RG7888, 3C8 and OX40L. Surface areas values were calculated by PISA (PDBePISA).

	DF004 VH	DF004 VL	RG7888 VH	RG7888 VL	3C8 VH	3C8 VL	OX40L
CRD1	43.2	0	0	0	0	0	239.93
CRD2	531.16	581.91	0	121.78	563.26	502.25	162.27
CRD3	0	0	573.91	338.45	0	0	42.57
Total	1156.27		1034.14		1065.51		444.77

Supplementary Figure

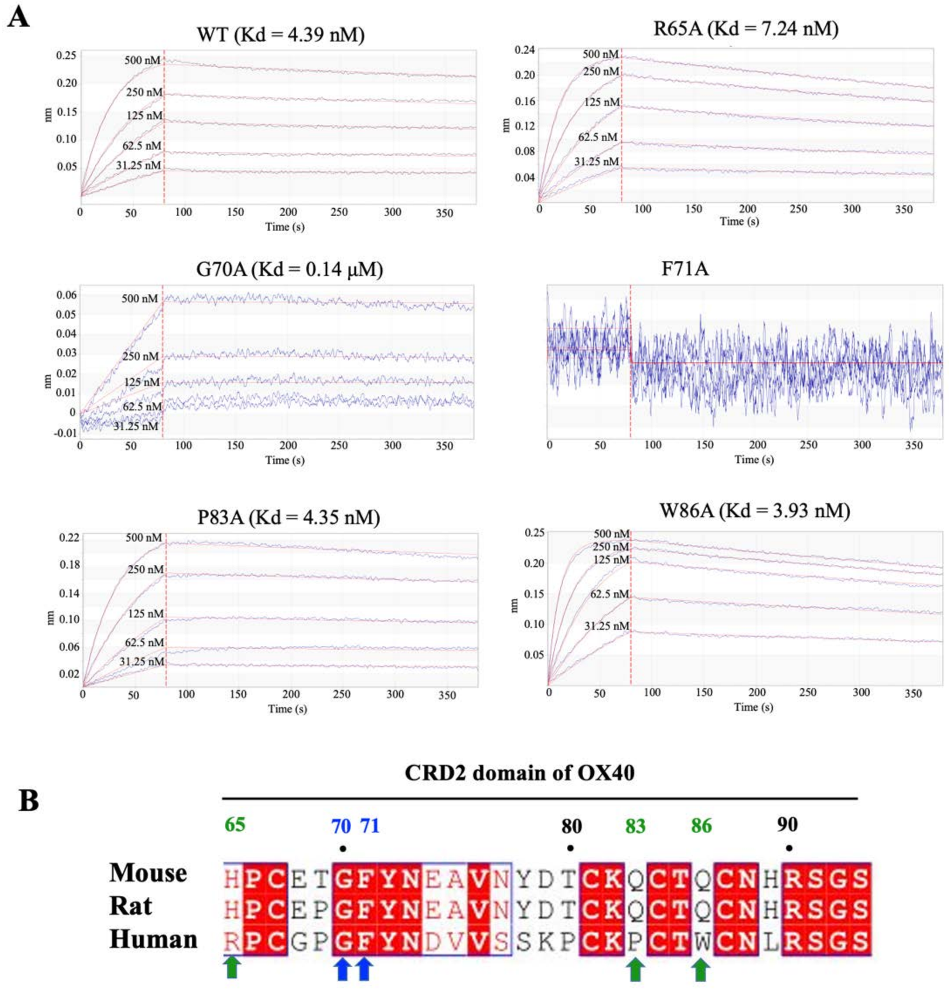

Figure S1. (**A**) Binding curve of DF004 with OX40 and its mutants. Serial dilutions of the OX40 mutants were applied to DF004 immobilized on an Fc sensor chip. The blue lines represented the measured data, and the red lines showed the fitted curves. (**B**) Sequence alignment of OX40 CRD2 domain among human, rat and mouse.
